# Impact of disease screening on awareness and management of hypertension and diabetes between 2011 and 2015: results from the China health and retirement longitudinal study

**DOI:** 10.1186/s12889-019-6753-x

**Published:** 2019-04-23

**Authors:** Chihua Li, L. H. Lumey

**Affiliations:** 1 Zhengzhou Central Hospital, Affiliated to Zhengzhou University, Henan, China; 2 Department of Epidemiology, Mailman School of Public Health, Columbia University, 722 W 168th St, 1617A, New York, NY 10032 USA

**Keywords:** CHARLS health survey, Aging population, Awareness, Management, Effective communication, Cardiovascular disease, Diabetes and hypertension

## Abstract

**Background:**

There has been a limited recognition of hypertension and diabetes in China which has compromised optimal treatment. It is not clear if a screening program implemented by a national health survey has improved awareness and management of these conditions.

**Methods:**

The China Health and Retirement Longitudinal Study (CHARLS) is an ongoing longitudinal health survey conducted since 2011 among Chinese people aged 45 years and older. Participants have been assessed every two years by interviews, physical examinations, and fasting glucose samples were taken in 2011. In 2013 and 2015, participants were asked about awareness and management of selected chronic diseases, and they first became aware of these conditions.

**Results:**

Of the 11,000+ participants screened in 2011, 4594 were identified with hypertension and 1703 with diabetes by medical examinations. Over 80% of the middle-aged and elderly Chinese diagnosed with hypertension and/or diabetes in 2011 reported in 2015 that they were unaware of the disease(s). Although some improvement was observed between 2011 and 2015, the main reason for the increase in awareness was a medical examination initiated by the study participant (over 75%), by their work unit or community (12–15%), and rarely (less than 3%) by the CHARLS examination. Participants with a rural household registration status and lower BMI were the most likely to be unaware and to remain unaware of their condition(s).

**Conclusions:**

Disease screening in CHARLS did not lead to significant improvements in awareness of hypertension and diabetes. Improvements should be made by the systematic feedback of screening results to survey participants and the monitoring of disease awareness over time. This will be essential to improve disease recognition and facilitate optimal management.

**Electronic supplementary material:**

The online version of this article (10.1186/s12889-019-6753-x) contains supplementary material, which is available to authorized users.

## Background

The rapidly increasing burden of chronic diseases in low and middle-income countries poses a major public health challenge across the world [1–3]. Recent studies also document the high prevalence of unrecognized diabetes [4, 5] and hypertension [6–9] among the Chinese population, and suggest that the low awareness of these conditions impedes their effective control and management [4–9]. Because hypertension and diabetes are important risk factors not only for cardiovascular but also for other diseases, their timely diagnosis and adequate management is crucial for the prevention of adverse health outcomes [10–17].

Although epidemiologic studies highlight the urgent need to improve awareness and treatment of hypertension and diabetes in China [4–9], they have not yet examined if screening for disease does improve participants’ awareness and disease management. In addition, it is not known how individuals with hypertension or diabetes in China first became aware of their condition(s) and what factors may have contributed to changes in awareness over time.

In this study, we examine the impact of a screening program on an individual’s awareness of hypertension and diabetes between 2011 and 2015 and the management of these conditions using data from the China Health and Retirement Longitudinal Study (CHARLS). We identify sources of disease awareness and factors that contribute to improved awareness. Our findings are relevant to help formulate more effective programs to improve awareness and management of these chronic conditions in China and other developing countries.

## Methods

### Study design and procedures

Starting in 2011, participants for the China Health and Retirement Longitudinal Study (CHARLS) were selected using a four-stage stratified cluster sampling method [18]. First, 150 county-level units from 28 provinces in China were sampled to represent a mix of urban and rural sittings with a wide variation of economic development. Then three primary sampling units (PSU), administrative villages in rural areas or residential neighborhoods in urban areas, were sampled within each county, resulting in a total of 450 villages/neighborhoods. Next, the dwellings in each PSU were mapped and 24 of the mapped households in each PSU were sampled for further studies. At every stage, further sampling was done by random selection. In each selected household, one individual aged 45 or older was invited to participate together with his or her spouse, if available. Overall, 17,708 participants from 10,257 households were interviewed; the overall household response rate was 80.5%.

The 2011 structured home interview was designed to collect information on basic demographics, family characteristics, participants’ health status, health care and insurance, and family economics, including employment and pensions, household and individual income and family expenditures. Participants were asked if they had ever been diagnosed with diabetes or hypertension. Later physical examinations included the measurement of height, weight, and blood pressure. These results were communicated to participants during the examination. Medically trained staff from the Chinese Center for Disease Control and Prevention (CDC) collected fasting venous blood samples using a standard protocol. Assays of the fasting glucose level and HbA1c were later carried out by central study laboratories in Beijing. Based on the informed consent, blood test results were mailed to participants three weeks after specimen collection [19]. However, we found no records how and when the diabetes blood test results were reported to study participants. Among those interviewed, 13,610 (76.9%) underwent a physical examination and 11,018 (62.2%) agreed to provide a fasting blood sample.

After 2011, study participants were re-interviewed every two years to monitor any changes over time in their health, economic, or social conditions. Participants were asked again if they had ever been diagnosed with hypertension or diabetes. Physical examinations were repeated in 2013 and 2015. Over 80% of participants interviewed in 2011 continued to participate in 2015, of this 85% underwent a physical examination in 2015. Blood samples were collected again in 2015, but to date, no assay results have been distributed for this year.

### Measures

For this study, hypertension was defined as (1) a mean systolic blood pressure (SBP) of 140 mmHg or higher; or (2) a mean diastolic blood pressure (DBP) of 90 mmHg or higher; and/or (3) a current use of anti-hypertensive medication [6]. Diabetes was defined as (1) a fasting plasma glucose level of 126 mg/dL (7.0 mmol/L) or higher; or (2) HbA1c concentration of 6.5% or higher; or (3) a self-report of doctor diagnosed diabetes [20].

Patients with hypertension or diabetes were defined as being aware of their condition if they reported they had ever been diagnosed. If so, they were asked how they became aware of the diagnosis: (1) by a physical examination for specific health conditions; (2) by a physical examination organized by their work unit or community; (3) by a physical examination organized by CHARLS; (4) or by other means.

Patients were then interviewed how their hypertension or diabetes was managed and/or monitored in the past 12 months and if they had received any specific treatments or medical advice. For hypertension, participants were asked if they had their blood pressure measured for at least once in the past year; if the treatments had included Chinese traditional medicine or Western medicine; and if medical recommendations had included weight control, exercise, diet or smoking control. Also, hypertensive participants whose SBP was lower than 140 mmHg and DBP was lower than 90 mmHg were considered to have their hypertension well-controlled. For diabetes, participants were asked if they had been given blood/urine glucose tests, or fundus examinations, or micro-albuminuria tests; if the treatments had included Chinese traditional medicine, Western medicine, or insulin injections; and if the medical recommendations had included weight control, exercise, diet, smoking control, or foot care.

Participants’ age was categorized into three groups (45–59, 60–69, and > =70 years), resident status was categorized into rural or urban Hukou (household registration status), and education was categorized as illiterate (cannot read or write), literate (received informal education or primary school education less than 5 years), primary school (5 to 6 years education), and junior school and above (received education for over 6 years). Per capita monthly household expenditure (PCE) (log-transformed) rather than income was used to define household resources following previous studies [5, 21]. Body mass index (BMI) was reported in three categories based on Asian-specific cutoffs: < 23 kg/m^2^ (Normal), 23 to < 27.5 kg/m^2^ (Overweight), and ≥ 27.5 kg/m^2^ (Obese) [22, 23].

### Statistical analysis

Demographic characteristics for CHARLS participants in 2011 and 2015 were tabulated separately for individuals with hypertension and diabetes, stratified by diagnosis awareness. National (‘weighted’) estimates were made using the survey sampling weights for each participant. These weights take the multi-level sampling design of the study into account and any missing information among study participants on anthropometric measures and/or blood assays. Analyses were performed with and without the survey sampling weights and the results were in close agreement. The main findings reported here reflect the original survey results. National (‘weighted’) estimates are presented in Additional file 1. Data collected in 2013 were also examined and are presented in Additional file 1.

Among individuals diagnosed with diabetes and/or hypertension in 2011 who were not aware of their condition, we compared the characteristics of those who were still not aware of their condition in 2015 with those who were. All analyses were conducted with StataSE 15 (StataCorp, College Station, USA).

## Results

Overall, full data were available for 9357 participants who completed surveys in both 2011 and 2015. Their baseline characteristics in 2011 are presented in Table 1. In 2011, 4594 (49.1%) of participants had a positive screening result for hypertension, 53.7% (*n* = 2466) of these were aware of their condition and 46.3% (*n* = 2128) were not, and 27.2% (*n* = 1251) did not have their hypertension well-controlled. Aware hypertension patients were more likely to be women, older, have urban Hukou status and have higher BMIs compared to those not aware. They were less likely to be current smokers or to be consuming alcohol. For diabetes, 1703 (18.2%) of participants had a positive screening result, and only 33.4% (*n* = 568) of these participants were aware of their condition and 66.6% (*n* = 1135) were not aware. Similar associations between hypertension awareness and diabetes awareness and demographic characteristics were observed.

**Table 1:** Characteristics of CHARLS participants by awareness of hypertension and diabetes in 2011

Characteristic	Total sample	Hypertension			Diabetes		
		Aware	Unaware	*P*-value	Aware	Unaware	*P*-value
No. (%)	9357	2466 (26.4)	2128 (22.7)	–	568 (6.1)	1135 (12.1)	–
Age, No. (%)							
45–59	5170 (55.3)	1085 (44.0)	1550 (54.0)	< 0.01	271 (47.7)	603 (53.1)	< 0.01
60–69	2711 (29.0)	881 (35.7)	556 (26.1)		210 (37.0)	333 (29.3)	
> =70	1476 (15.8)	500 (20.3)	422 (19.8)		87 (15.3)	199 (17.5)	
Sex, No. (%)							
Men	4310 (46.1)	1077 (43.7)	1029 (48.4)	< 0.01	248 (43.7)	540 (47.6)	0.13
Women	5047 (53.9)	1389 (56.3)	1099 (51.6)		320 (56.3)	595 (52.4)	
Hukou status, No. (%)							
Urban	1679 (17.9)	551 (22.3)	395 (18.6)	< 0.01	177 (31.2)	212 (18.7)	< 0.01
Rural	7678 (82.1)	1915 (77.7)	1733 (81.4)		391 (68.8)	923 (81.3)	
Education, No. (%)							
Illiterate	2682 (28.7)	734 (29.8)	651 (30.6)	0.09	138 (24.3)	329 (29.0)	0.03
Literate (< 5 yrs)	1723 (18.4)	473 (19.2)	347 (16.3)		99 (17.4)	232 (20.4)	
Primary school (5–6 yrs)	2064 (22.1)	547 (22.2)	487 (22.9)		130 (22.9)	226 (19.9)	
Junior school + (> 6 yrs)	2888 (30.9)	712 (28.9)	643 (30.2)		201 (35.4)	348 (30.7)	
Log (PCE^a^), No. (%)							
Bottom tertile (< 170 rmb)	3119 (33.3)	821 (33.3)	723 (34.0)	0.89	162 (28.6)	384 (33.8)	< 0.01
Middle tertile (170–519 rmb)	3119 (33.3)	810 (32.8)	693 (32.6)		163 (28.8)	396 (34.9)	
Top tertile (> 519 rmb)	3119 (33.3)	835 (33.9)	712 (33.4)		243 (42.6)	355 (31.3)	
Smoking status, No. (%)							
Current	2660 (28.4)	579 (23.5)	616 (29.0)	< 0.01	121 (21.3)	340 (30.0)	< 0.01
Ever	813 (8.7)	280 (11.4)	153 (7.2)		75 (13.2)	111 (9.8)	
None	5884 (62.9)	1607 (65.2)	1359 (63.9)		372 (65.5)	684 (60.3)	
Past-year alcohol drinking, No. (%)							
Yes	3058 (32.7)	675 (27.4)	767 (36.0)	< 0.01	154 (27.1)	396 (34.9)	< 0.01
No	6299 (67.3)	1791 (72.6)	1361 (64.0)		414 (72.9)	739 (65.1)	
BMI, No. (%)							
< 23 (Normal)	4482 (47.9)	754 (30.6)	1044 (49.1)	< 0.01	145 (25.5)	472 (41.6)	< 0.01
23- < 27.5 (Overweight)	3528 (37.7)	1065 (43.2)	775 (36.4)		258 (45.5)	434 (38.2)	
> =27.5 (Obese)	1347 (14.4)	647 (26.2)	309 (14.6)		165 (29.0)	229 (20.2)	

^a^*PCE* Per capita monthly household expenditure

In 2015, the majority of unaware hypertension patients (1704 of 2128, 80.1%) and diabetes patients (972 of 1135, 85.6%) were still not aware of their condition (Additional file 1: Table S1), in spite of their positive screening results in 2011. And 25.7% (*n* = 1180) of those 4594 participants with positive hypertensive screening results in 2011 did not have their hypertension well-controlled in 2015. The disease awareness for hypertension and diabetes was somewhat higher in 2015 compared to 2011. Similar associations with demographic characteristics were observed in 2011 and 2015. Findings related to the awareness of hypertension and diabetes were similar in 2013 and 2015, but any changes relative to the 2011 survey were not yet as pronounced in 2013 as in 2015 (Additional file 1: Table S2).

**Fig. 1:**
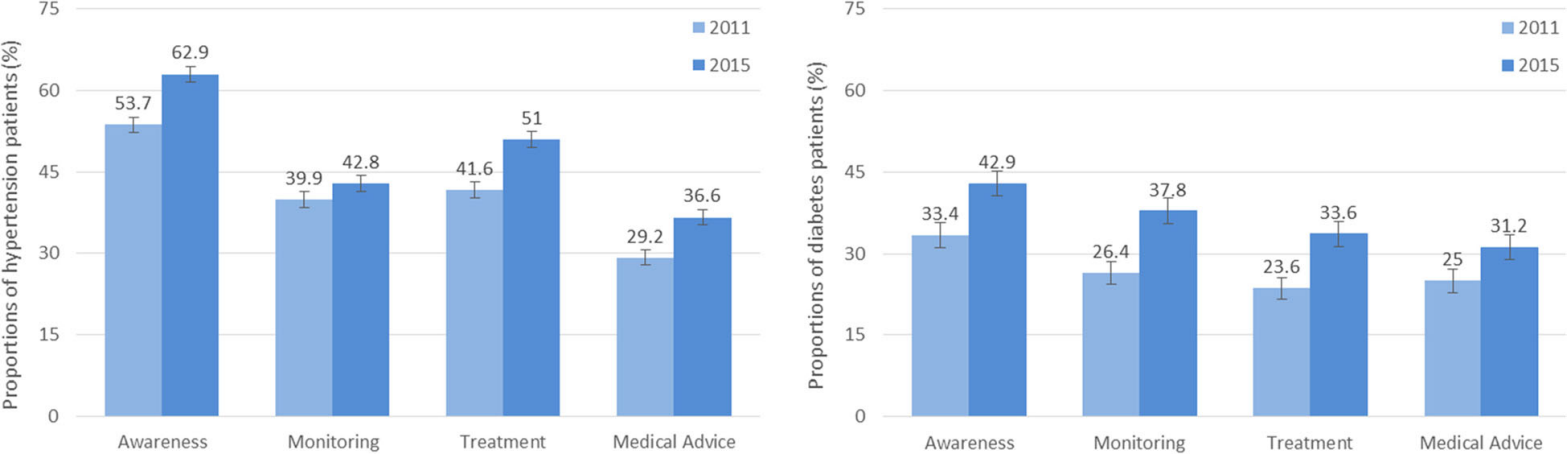
Awareness and management of hypertension and diabetes among CHARLS participants in 2011 and 2015. Awareness: among all participants with hypertension/diabetes, the proportion that were aware of their condition. Monitoring: among participants with hypertension, the proportion that had blood pressure measured at least once in the past 12 months; among participants with diabetes, the proportion that received blood/urine glucose tests, or fundus examinations, or micro-albuminuria tests at least once in the past 12 months. Treatment: among all participants with hypertension/diabetes, the proportion that received current medical treatment for this chronic condition in the past 12 months. Medical advice: among all participants with hypertension/diabetes, the proportion that received medical recommendations from their doctor(s) in the past 12 months

Some improvements in awareness and management of diabetes and hypertension were observed between 2011 and 2015 (Fig. 1). Among participants who had a positive screening result for hypertension in 2011, the proportion aware of this condition increased from 53.7 to 62.9% (17.1% improvement), having blood pressure monitored at least once in the past 12 months increased from 39.9 to 42.8% (7.3% improvement), having received current medication increased from 41.6 to 51.0% (22.6% improvement), and having received medical advice from doctors at least once in the past 12 months increased from 29.2 to 36.6% (20.2% improvement).

Among participants who had a positive screening result for diabetes in 2011, the proportion aware of this condition increased from 33.4 to 42.9% (28.4% improvement), having received tests to monitor their diabetic condition at least once in the past 12 months increased from 26.4 to 37.8% (43.2% improvement), having received current medication increased from 23.6 to 33.6% (42.4% improvement), and having received medical advice from doctors at least once in the past 12 months increased from 25.0 to 31.2% (24.8% improvement).

Above improvements, especially in disease awareness, can be mainly attributed to sources other than CHARLS screening (Table 2). Among 2890 hypertension patients who were aware of their condition in 2015, 76.8% reported that they learned the diagnosis (either before or after 2011) from a physical examination (unrelated to CHARLS screening) for specific health conditions, 11.9% from a physical examination organized by their work unit or community, and only 2.6% from the screening organized by CHARLS. A similar pattern was observed among the 731 aware diabetes patients. From the CHARLS documentation, it is not clear if the physical examination for specific health conditions was prompted by a visit to medical doctors for other ailments or not.

**Table 2:** Source of disease awareness among disease aware patients in 2015

	Aware hypertension patients	Aware diabetes patients
No.	2890	731
Source of awareness, No. (%)		
Physical examination for specific health conditions	2220 (76.8)	557 (76.2)
Physical examination organized by work unit or community	344 (11.9)	107 (14.6)
Physical examination organized by CHARLS survey	75 (2.6)	15 (2.1)
Others or not clear	251 (8.7)	52 (7.1)

Baseline characteristics of hypertension and diabetes patients who were unaware of their condition in 2011 are compared between those who in 2015 became aware of their condition or not (Table 3). Among the 2128 participants with unknown hypertension and 1135 participants with unknown diabetes in 2011, those with urban Hukou status and higher BMI were more likely to become aware of their condition in 2015.

**Table 3:** Comparisons of participants who became aware of their disease condition and who remained unaware in 2015

Awareness in 2015	Unaware hypertension patients in 2011 (*n* = 2128)			Unaware diabetes patients in 2011 (*n* = 1135)		
	Yes	No	*p*-value	Yes	No	*p*-value
No. (%)	424	1704	–	163	972	–
Age, No. (%)						
45–59	210 (49.5)	940 (55.2)	< 0.01	90 (55.2)	513 (52.8)	0.80
60–69	142 (33.5)	414 (24.3)		47 (28.8)	286 (29.4)	
> =70	72 (17.0)	350 (20.5)		26 (16.0)	173 (17.8)	
Sex, No. (%)						
Men	213 (50.2)	816 (47.9)	0.39	58 (35.6)	482 (49.6)	< 0.01
Women	211 (49.8)	888 (52.1)		105 (64.4)	490 (50.4)	
Hukou status, No. (%)						
Urban	158 (37.3)	337 (19.8)	< 0.01	40 (24.5)	172 (17.7)	0.04
Rural	266 (62.7)	1367 (80.2)		123 (75.5)	800 (82.3)	
Education, No. (%)						
Illiterate	132 (31.1)	519 (30.5)	0.43	47 (28.8)	282 (29.0)	0.44
Literate (< 5 yrs)	69 (16.3)	278 (16.3)		28 (17.2)	204 (21.0)	
Primary school (5–6 yrs)	107 (25.2)	380 (22.3)		30 (18.4)	196 (20.2)	
Junior school + (> 6 yrs)	116 (27.4)	527 (30.9)		58 (35.6)	290 (29.8)	
Log (PCE^a^), No. (%)						
Bottom tertile (< 261 rmb)	155 (36.6)	568 (33.3)	0.24	57 (35.0)	327 (33.7)	0.44
Middle tertile (261–702 rmb)	141 (33.3)	552 (32.4)		50 (30.6)	347 (35.7)	
Top tertile (> 702 rmb)	128 (30.1)	584 (34.3)		56 (34.4)	298 (30.7)	
Smoking status, No. (%)						
Current	41 (9.7)	112 (6.6)	0.04	16 (9.8)	95 (9.8)	0.08
Ever	130 (30.7)	486 (28.5)		37 (22.7)	303 (31.2)	
None	253 (59.7)	1106 (64.9)		110 (67.5)	574 (59.1)	
Past-year alcohol drinking, No. (%)						
Yes	157 (37.0)	610 (35.8)	0.64	43 (26.4)	353 (36.3)	0.01
No	267 (63.0)	1094 (64.2)		120 (73.6)	619 (63.7)	
BMI, No. (%)						
< 23 (Normal)	178 (42.0)	879 (51.6)	< 0.01	29 (17.8)	442 (45.5)	< 0.01
23- < 27.5 (Overweight)	169 (39.8)	600 (35.2)		81 (49.6)	353 (36.3)	
> =27.5 (Obese)	77 (18.2)	225 (13.2)		53 (32.6)	177 (18.2)	

^a^*PCE* Per capita monthly household expenditure

National estimates based on the survey findings were obtained by using the survey sampling weights, extending our findings to the entire Chinese population aged 45 and older (Additional file 1: Table S3). Over 80% (83.4 of 101.7 million) of all middle-aged and elderly Chinese with undiagnosed hypertension in 2011 are likely to still be unaware of their condition in 2015, and over 85% (42.3 of 48.1 million) of those with undiagnosed diabetes.

## Discussion

Our findings show that CHARLS participants diagnosed with hypertension and/or diabetes showed some improvement in disease awareness and between 2011 and 2015. Many more reported they were unaware of their condition, four years after the health survey. Improvements in disease awareness came mostly from a physical examination for specific health conditions unrelated to CHARLS (over 75%) or an examination organized by a work unit or community (12–15%). The CHARLS screening in 2011 contributed less than 3% to the improvement in disease awareness.

Several reasons could explain the limited increase in reported disease awareness in 2015. First, some participants may not have received the physical examination and blood test results from the 2011 survey; second, they may have not understood the results; or third, they may have forgotten the results. Alternatively, they may have been unwilling to recognize that they had been diagnosed with diabetes or hypertension and also failed to take appropriate measures for disease management. This will need further study.

According to available CHARLS documentation (Additional file 2), the blood test results included cell counts processed by local Centers for Disease Control (CDC) units. These were mailed to addresses provided by study participants. No information is provided in CHARLS documentation on the nature of any health consultations, either what specific clarifications or medical advice might have been given at the time the physical examination or when diabetes blood test results were reported to study participants. Further analyses including glucose and HbA1c assays were later carried out by central study laboratories in Beijing. The CHARLS documents make no mention of communication of further diabetes testing to study participants. However, based on the follow-up interviews in 2013 and 2015, we know that only a small proportion of CHARLS participants reported that they learned about their hypertension and/or diabetes status by this screening. Future CHARLS protocols will need to clarify how a systematic and effective feedback of screening results to survey participants will be accomplished. In CHARLS, there were still many participants who did not have any hypertension or diabetes treatment in 2015 despite being informed of their condition in 2011. It will be interesting to explore reasons for why those participants did not have treatment in future studies.

Our findings on disease awareness and management were in agreement with results from earlier cross-sectional studies [3, 4, 9, 24]. This study further demonstrates through a well-conducted follow-up study that disease screening may only have limited impact on improving awareness of disease. This study highlights that conducting a good follow-up study does not necessarily translate into imparting adequate or appropriate information on hypertension and diabetes status to study participants. It appears that more effective follow-up procedures will be needed to improve the awareness and management of diabetes and hypertension after CHARLS examinations. This will require specific attention to the distribution of test results, counseling, and the provision of health care services.

Documenting changes of disease awareness and management over time in surveys is important, as this could also help identify individuals at risk who remain unaware of their conditions. Overall, we found that CHARLS participants with rural Hukou status are less likely to be aware they have diabetes or hypertension and were less likely to benefit from the CHARLS screening. Comparing patients with hypertension and diabetes, we noticed that patients unaware of one condition were also more likely to be unaware of the other, suggesting a cluster of unawareness.

Extensive evidence has shown that blood pressure control and blood glucose control can significantly reduce adverse cardiovascular events [10–13, 25, 26] and prevent other adverse health outcomes [14–17]. For example, one clinical trial has shown that even a small reduction in blood pressure could significantly reduce the risk of heart failure, stroke, and myocardial infarction [26]. Therefore targeted interventions should be considered in individuals with hypertension or diabetes identified by the CHARLS survey. It must be assured that they have adequate access to relevant health information and opportunities to take appropriate actions.

Since CHARLS is one of the cohort studies that follows the design of the Health and Retirement Study (HRS) in the United States, we reviewed the reporting of physical examination results in other studies of the HRS family. Among the 18 HRS family studies worldwide, most had collected blood samples [27, 28]. Some [29, 30] but not all studies reported test results back to study participants and some [18, 31–33] provided specific advice to study participants that they seek a health consultation. As an example, the HRS protocol lists specific procedures for the reporting of blood test results to study participants. In all, the protocol lists 27 items, including complete blood cell counts, fasting glucose, and HbA1c [29, 34, 35]. In the English Longitudinal Study of Aging (ELSA), all blood test results from study participants are reported to their primary care physicians (‘general practitioners’) [32]. To the best of our knowledge, however, no studies from the HRS family have at this point systematically evaluated the relation between a positive disease finding in earlier survey rounds and changes in disease awareness in subsequent examinations. The observed variations in the reporting of screening results across HRS studies could reflect cultural differences in participant expectation and the role and function of health surveys. This needs further exploration.

Our findings raise important questions about the effective communication of screening results not only in CHARLS but also in other health surveys. In CHARLS, the change in awareness was similar for hypertension and diabetes. One possible explanation is that screening, in either health surveys or other contexts, is seen simply as an isolated process and not as a tool for follow-up, treatment, or referral. In China, individuals were too often expected to take actions by themselves after receiving the screening results [36]. It is important therefore that increased efforts are made to make sure that participants do understand the medical examination results and are motivated to access the appropriate health services where needed. As part of these efforts, separate studies should be initiated to identify effective tools for the communication of screening results. Several methods, including follow-up phone calls or even direct medical referrals, could be explored to address this problem. In general, we recommend the systematic monitoring of disease awareness over time in longitudinal studies so that communication procedures can be improved where needed. Thereby the impact of these and other screening programs on participants’ health can be maximized.

## Conclusions

While we observed an increase in hypertension and diabetes awareness over time in the CHARLS survey, the persistent limited awareness of these conditions remains a major public health concern. Our findings suggest a ‘failure to act on the findings from screening’ [36] as also reported for a previous hypertension program. To be more effective, disease screening programs need to pay attention not only to effective diagnosis but also to the systematic evaluation of measures related to disease awareness and management. If awareness remains low, communications with study participants will need to be improved. The CHARLS findings to date suggest that populations with rural Hukou status are most likely to benefit most from targeted screening studies that incorporate these insights.

## Additional files

Additional file 1: **Table S1** Characteristics of CHARLS participants by awareness of hypertension and diabetes in 2015. **Table S2.** Characteristics of CHARLS participants by awareness of hypertension and diabetes in 2013. **Table S3.** National estimates by population characteristics and awareness of hypertension and diabetes in 2011 and 2015 (numbers are in million). (DOCX 24 kb)

Additional file 2: Supplementary Text. CHARLS documentation on the nature of the feedback provided to its participants. (DOCX 13 kb)

## Abbreviation

CHARLS: China Health and Retirement Longitudinal Study
