# Supplementary material for: Impact of disease screening on awareness and management of hypertension and diabetes between 2011 and 2015: results from the China health and retirement longitudinal study
Source: BMC Public Health. 2019 Apr 23;19:421. doi: 10.1186/s12889-019-6753-x (PMC6480849; doi:10.1186/s12889-019-6753-x)
Supplement: Supplementary file 1 — Table S1 Characteristics of CHARLS participants by awareness of hypertension and diabetes in 2015. Table S2. Characteristics of CHARLS participants by awareness of hypertension and diabetes in 2013. Table S3. National estimates by population characteristics and awareness of hypertension and diabetes in 2011 and 2015 (numbers are in million). (DOCX 24 kb) [file 12889_2019_6753_MOESM1_ESM.docx]

| **Characteristic** | **Total sample** | **Hypertension** | | | **Diabetes** | | |
| --- | --- | --- | --- | --- | --- | --- | --- |
|  |  | **Aware** | **Unaware** | **P-value** | **Aware** | **Unaware** | **P-value** |
| **No. (%)** | 9357 | 2890 (30.9) | 1704 (18.2) | - | 731 (7.8) | 972 (10.4) | - |
| **Age, No. (%)** | | | | | | | |
| **45-59** | 3600 (38.5) | 822 (28.4) | 650 (38.2) | <0.01 | 234 (32.0) | 340 (35.0) | 0.10 |
| **60-69** | 3477 (37.2) | 1185 (41.0) | 570 (33.5) |  | 315 (43.1) | 369 (38.0) |  |
| **>=70** | 2280 (24.4) | 883 (30.6) | 484 (28.4) |  | 182 (24.9) | 236 (27.1) |  |
| **Sex, No. (%)** | | | | | | | |
| **Men** | 4310 (46.1) | 1290 (44.6) | 816 (47.9) | 0.03 | 306 (41.9) | 482 (49.6) | <0.01 |
| **Women** | 5047 (53.9) | 1600 (55.4) | 888 (52.1) |  | 425 (58.1) | 490 (50.4) |  |
| **Hukou status, No. (%)** | | | | | | | |
| **Urban** | 1947 (20.8) | 609 (21.1) | 337 (19.8) | 0.29 | 217 (29.7) | 172 (17.7) | <0.01 |
| **Rural** | 7410 (79.2) | 2281 (78.9) | 1367 (80.2) |  | 514 (70.3) | 514 (70.3) |  |
| **Education, No. (%)** | | | | | | | |
| **Illiterate** | 2593 (27.7) | 838 (29.0) | 507 (29.8) | 0.10 | 178 (24.4) | 276 (28.4) | 0.05 |
| **Literate (<5 yrs)** | 1740 (18.6) | 550 (19.0) | 282 (16.6) |  | 132 (18.1) | 200 (20.6) |  |
| **Primary school (5-6 yrs)** | 2087 (22.3) | 663 (22.9) | 379 (22.2) |  | 158 (21.6) | 198 (20.4) |  |
| **Junior school+ (>6 yrs)** | 2937 (31.4) | 839 (29.0) | 536 (31.5) |  | 263 (36.0) | 298 (30.7) |  |
| **Log(PCE*), No. (%)** | | | | | | | |
| **Bottom tertile (<261 rmb)** | 3119 (33.3) | 957 (33.1) | 602 (35.3) | 0.10 | 188 (25.7) | 372 (38.3) | <0.01 |
| **Middle tertile (261-702 rmb)** | 3119 (33.3) | 1007 (34.9) | 520 (30.5) |  | 234 (32.0) | 303 (31.2) |  |
| **Top tertile (>702 rmb)** | 3119 (33.3) | 926 (32.0) | 582 (34.2) |  | 309 (42.3) | 297 (30.6) |  |
| **Smoking status, No. (%)** | | | | | | | |
| **Current** | 2457 (26.3) | 636 (22.0) | 448 (26.3) | <0.01 | 137 (18.7) | 289 (29.7) | <0.01 |
| **Ever** | 1016 (10.9) | 394 (13.6) | 150 (8.8) |  | 112 (15.3) | 109 (11.2) |  |
| **None** | 5884 (62.9) | 1860 (64.4) | 1106 (64.9) |  | 482 (65.9) | 574 (59.1) |  |
| **Past-year alcohol drinking, No. (%)** | | | | | | | |
| **Yes** | 2353 (25.2) | 623 (21.6) | 501 (29.4) | <0.01 | 138 (18.9) | 238 (29.1) | <0.01 |
| **No** | 7004 (74.9) | 2267 (78.4) | 1203 (70.6) |  | 593 (81.1) | 689 (70.9) |  |
| **BMI, No. (%)** | | | | | | | |
| **<23 (Normal)** | 4108 (43.9) | 847 (29.3) | 814 (47.8) | <0.01 | 202 (27.6) | 416 (42.8) | <0.01 |
| **23-<27.5 (Overweight)** | 1948 (40.1) | 1269 (43.9) | 646 (37.9) |  | 350 (47.9) | 386 (39.7) |  |
| **>=27.5 (Obese)** | 3301 (16.0) | 774 (26.8) | 244 (14.3) |  | 179 (24.5) | 170 (36.9) |  |

Supplementary Table 1. Characteristics of CHARLS participants by awareness of hypertension and diabetes in 2015

* **PCE:** Per capita monthly household expenditure.

| **Characteristic** | **Total sample** | **Hypertension** | | | **Diabetes** | | |
| --- | --- | --- | --- | --- | --- | --- | --- |
|  |  | **Aware** | **Unaware** | **P-value** | **Aware** | **Unaware** | **P-value** |
| **No. (%)** | 9357 | 2707 (28.9) | 1892 (20.2) | - | 632 (6.8) | 1071 (11.4) | - |
| **Age, No. (%)** | | | | | | | |
| **45-59** | 4380 (46.8) | 922 (34.1) | 890 (47.0) | <0.01 | 252 (39.9) | 490 (45.8) | <0.01 |
| **60-69** | 3092 (33.0) | 963 (35.6) | 560 (29.6) |  | 276 (43.7) | 350 (32.7) |  |
| **>=70** | 1885 (20.1) | 822 (30.4) | 442 (16.3) |  | 104 (16.5) | 231 (21.6) |  |
| **Sex, No. (%)** | | | | | | | |
| **Men** | 4310 (46.1) | 1210 (44.6) | 932 (49.3) | 0.02 | 273 (43.2) | 503 (47.0) | 0.10 |
| **Women** | 5047 (53.9) | 1497 (55.4) | 960 (50.7) |  | 359 (56.8) | 568 (53.0) |  |
| **Hukou status, No. (%)** | | | | | | | |
| **Urban** | 1749 (18.7) | 581 (21.5) | 367 (19.4) | 0.15 | 190 (30.1) | 190 (17.7) | <0.01 |
| **Rural** | 7608 (81.3) | 2126 (78.5) | 1525 (80.6) |  | 442 (69.9) | 881 (82.3) |  |
| **Education, No. (%)** | | | | | | | |
| **Illiterate** | 2615 (27.9) | 791 (29.2) | 562 (29.7) | 0.23 | 166 (26.3) | 297 (27.7) | 0.15 |
| **Literate (<5 yrs)** | 1730 (18.5) | 514 (19.0) | 310 (16.4) |  | 121 (19.1) | 221 (20.6) |  |
| **Primary school (5-6 yrs)** | 2077 (22.2) | 597 (22.1) | 422 (22.3) |  | 141 (22.3) | 205 (19.1) |  |
| **Junior school+ (>6 yrs)** | 2935 (31.4) | 805 (29.7) | 598 (31.6) |  | 204 (32.3) | 348 (32.5) |  |
| **Log(PCE*), No. (%)** | | | | | | | |
| **Bottom tertile (<220 rmb)** | 3119 (33.3) | 933 (34.5) | 688 (36.4) | 0.28 | 171 (27.1) | 379 (35.4) | <0.01 |
| **Middle tertile (220-638 rmb)** | 3119 (33.3) | 990 (36.6) | 610 (32.2) |  | 193 (30.5) | 353 (33.0) |  |
| **Top tertile (>638 rmb)** | 3119 (33.3) | 784 (29.0) | 594 (31.4) |  | 268 (42.4) | 339 (31.7) |  |
| **Smoking status, No. (%)** | | | | | | | |
| **Current** | 2550 (27.3) | 610 (22.5) | 584 (30.9) | <0.01 | 131 (20.7) | 311 (29.0) | <0.01 |
| **Ever** | 923 (9.9) | 362 (13.4) | 152 (8.0) |  | 102 (16.1) | 110 (10.3) |  |
| **None** | 5884 (62.9) | 1735 (64.1) | 1156 (61.1) |  | 399 (63.1) | 650 (60.7) |  |
| **Past-year alcohol drinking, No. (%)** | | | | | | | |
| **Yes** | 2769 (29.6) | 661 (24.4) | 611 (32.3) | <0.01 | 145 (22.9) | 289 (27.0) | <0.01 |
| **No** | 6588 (70.4) | 2046 (75.6) | 1281 (67.7) |  | 487 (77.1) | 782 (73.0) |  |
| **BMI, No. (%)** | | | | | | | |
| **<23 (Normal)** | 4238 (45.3) | 793 (29.3) | 920 (48.6) | <0.01 | 181 (28.6) | 451 (42.1) | <0.01 |
| **23-<27.5 (Overweight)** | 2860 (30.6) | 1156 (42.7) | 691 (36.5) |  | 290 (45.9) | 413 (38.6) |  |
| **>=27.5 (Obese)** | 2259 (24.1) | 758 (28.0) | 281 (14.6) |  | 161 (25.5) | 207 (19.3) |  |

Supplementary Table 2. Characteristics of CHARLS participants by awareness of hypertension and diabetes in 2013

* **PCE:** Per capita monthly household expenditure.

| **Characteristic** | **Hypertension** | | | | **diabetes** | | | |
| --- | --- | --- | --- | --- | --- | --- | --- | --- |
|  | **2011** | | **2015** | | **2011** | | **2015** | |
|  | **Aware** | **Unaware** | **Aware** | **Unaware** | **Aware** | **Unaware** | **Aware** | **Unaware** |
| **No. (in million)** | 112.5 | 101.7 | 130.7 | 83.4 | 27.0 | 48.1 | 32.8 | 42.3 |
| **Age** | | | | | | | | |
| **45-59** | 50.7 | 56.4 | 36.5 | 32.6 | 12.2 | 25.1 | 10.9 | 15.3 |
| **60-69** | 40.6 | 26.7 | 53.8 | 28.8 | 9.6 | 13.4 | 13.8 | 17.0 |
| **>=70** | 21.2 | 18.5 | 40.4 | 22.0 | 5.2 | 9.7 | 8.1 | 10.4 |
| **Sex** | | | | | | | | |
| **Men** | 48.3 | 49.8 | 58.9 | 40.1 | 12.6 | 23.3 | 14.1 | 21.2 |
| **Women** | 64.2 | 51.9 | 71.8 | 43.3 | 14.4 | 24.8 | 18.7 | 21.1 |
| **Hukou status** | | | | | | | | |
| **Urban** | 24.6 | 18.0 | 28.1 | 16.3 | 9.0 | 8.5 | 9.8 | 7.1 |
| **Rural** | 87.9 | 83.7 | 102.6 | 67.1 | 18.0 | 39.6 | 23.0 | 35.2 |
| **Education** | | | | | | | | |
| **Illiterate** | 33.9 | 32.0 | 37.8 | 24.6 | 7.1 | 14.5 | 7.8 | 11.9 |
| **Literate (<5 yrs)** | 21.7 | 17.4 | 25.0 | 13.8 | 5.2 | 10.2 | 6.4 | 8.8 |
| **Primary school (5-6 yrs)** | 24.5 | 23.2 | 29.8 | 18.6 | 6.6 | 9.0 | 7.8 | 9.1 |
| **Junior school+ (>6 yrs)** | 32.4 | 29.1 | 38.2 | 26.4 | 8.1 | 14.3 | 11.4 | 12.5 |
| **Log(PCE*)** | | | | | | | | |
| **Bottom tertile** | 37.8 | 34.7 | 43.0 | 29.6 | 7.4 | 16.6 | 8.2 | 16.5 |
| **Middle tertile** | 36.8 | 32.4 | 46.4 | 25.2 | 7.8 | 16.2 | 10.9 | 13.1 |
| **Top tertile** | 37.9 | 34.6 | 41.3 | 28.6 | 11.8 | 15.3 | 13.7 | 12.7 |
| **Smoking status** | | | | | | | | |
| **Current** | 27.5 | 30.0 | 27.3 | 23.9 | 5.7 | 15.2 | 6.0 | 13.1 |
| **Ever** | 12.8 | 7.4 | 19.0 | 6.8 | 3.5 | 4.9 | 5.3 | 5.2 |
| **None** | 72.2 | 64.3 | 84.4 | 52.7 | 17.8 | 28.0 | 21.5 | 24.1 |
| **Past-year alcohol drinking** | | | | | | | | |
| **Yes** | 32.9 | 38.4 | 29.5 | 24.6 | 7.2 | 16.9 | 6.3 | 12.2 |
| **No** | 78.5 | 63.3 | 101.2 | 50.5 | 19.8 | 31.2 | 26.5 | 30.1 |
| **BMI** | | | | | | | | |
| **<23 (Normal)** | 34.0 | 51.1 | 38.2 | 40.7 | 6.7 | 19.7 | 9.2 | 17.8 |
| **23-<27.5 (Overweight)** | 47.8 | 36.5 | 58.2 | 31.4 | 12.1 | 19.0 | 15.6 | 16.1 |
| **>=27.5 (Obese)** | 30.7 | 14.1 | 34.3 | 11.3 | 8.2 | 9.4 | 8.0 | 8.4 |

Supplementary Table 3. National estimates by population characteristics and awareness of hypertension and diabetes in 2011 and 2015 (numbers are in million)

* **PCE:** Per capita monthly household expenditure.
