# Supplementary material for: Impact of disease screening on awareness and management of hypertension and diabetes between 2011 and 2015: results from the China health and retirement longitudinal study
Source: BMC Public Health. 2019 Apr 23;19:421. doi: 10.1186/s12889-019-6753-x (PMC6480849; doi:10.1186/s12889-019-6753-x)
Supplement: Supplementary file 2 — Supplementary Text. CHARLS documentation on the nature of the feedback provided to its participants. (DOCX 13 kb) [file 12889_2019_6753_MOESM2_ESM.docx]

**Supplementary Text. CHARLS documentation on the nature of the feedback provided to its participants**

Blood field work plan (Chinese version): (page 13)

在采血结束后的2周内，县CDC告知采血对象血常规检测结果。

Two weeks after the blood collection, CDC at the county level will notify the study participants of blood test results.

Consent form 2011 (English version): (page 1)

Roughly after three weeks of the blood tests we will inform you to obtain the blood routine examination results and the need for follow-up health counseling.

Consent form 2011 (Chinese version): (page 1)

大约三个星期后我们会通知您获得您的血常规化验结果和接受进一步的健康咨询。

We will inform you ... to receive further health consultation

In 2011 CHARLS questionnaire: (page 17)

AIK5. Could you give us your mailing address, so we can send you blood test result later?

能否告诉我们您家的邮寄地址，这样我们能够将血检结果寄给您。

Further examination of the CHARLS blood collection procedures indicates that these blood test results only included the blood cell counts: in the CHARLS Users’ Guide for the 2011-2012 National Baseline Blood Data (pages 3-4) it is stated specifically that three tubes of blood are to be collected: The first for a complete blood count (CBC). The second for glucose and lipid assays after separation of plasma and coat. And the third for HbA1c. The first tube was analyzed locally within hours at county CDC stations or town/village health centers. The other two tubes were locally processed, stored, and frozen, and shipped to the China CDC in Beijing within two weeks for deep freezing and storage until further assays at China CDC or Capital Medical University in Beijing. No mention is made of retesting of glucose or HbA1c results although this could have been part of internal laboratory control procedures.

From the description above we infer that the 3-week blood test results could only have included selected blood cell counts locally processed by CDC labs and not later analyses for glucose or HbA1c centrally processed in Beijing.

We also tried to examine which setting the health consultation took place, how many of the participants attended the health consultation, was medication prescribed during this health consultation, or were people referred to medical care. To answer these questions, we refer to the following items from the 2014 CHARLS life history wave (extra wave) questionnaire (page 120):

HS004 Did you take the blood test by our project team in 2011?

HS005 Have you ever received the blood test report?

HS006 I’m very sorry, due to some reasons, the blood test report could not be delivered to your home. Do you need us to provide the blood test report again?

The responses to these questions do not yet show up in any of the CHARLS databases. These variables are still missing from variables distributed to date. We therefore have no further information on whether blood test reports were received (or on the nature of health consultations, if any).
